# A Stable Biologically Motivated Learning Mechanism for Visual Feature Extraction to Handle Facial Categorization

**DOI:** 10.1371/journal.pone.0038478

**Published:** 2012-06-13

**Authors:** Karim Rajaei, Seyed-Mahdi Khaligh-Razavi, Masoud Ghodrati, Reza Ebrahimpour, Mohammad Ebrahim Shiri Ahmad Abadi

**Affiliations:** 1 Department of Mathematics and Computer Science, Amirkabir University of Technology, Tehran, Iran; 2 School of Cognitive Sciences (SCS), Institute for Research in Fundamental Sciences (IPM), Niavaran, Tehran, Iran; 3 Department of Mathematics, Statistics and Computer Science, University of Tehran, Tehran, Iran; 4 MRC Cognition and Brain Sciences Unit, Cambridge University, Cambridge, United Kingdom; 5 Department of Biomedical Engineering, Faculty of Engineering, Shahed University, Tehran, Iran; 6 Brain & Intelligent Systems Research Lab (BISLab), Department of Electrical and Computer Engineering, Shahid Rajaee Teacher Training University, Tehran, Iran; University of Leuven, Belgium

## Abstract

The brain mechanism of extracting visual features for recognizing various objects has consistently been a controversial issue in computational models of object recognition. To extract visual features, we introduce a new, biologically motivated model for facial categorization, which is an extension of the Hubel and Wiesel simple-to-complex cell hierarchy. To address the synaptic stability versus plasticity dilemma, we apply the Adaptive Resonance Theory (*ART*) for extracting informative intermediate level visual features during the learning process, which also makes this model stable against the destruction of previously learned information while learning new information. Such a mechanism has been suggested to be embedded within known laminar microcircuits of the cerebral cortex. To reveal the strength of the proposed visual feature learning mechanism, we show that when we use this mechanism in the training process of a well-known biologically motivated object recognition model (the *HMAX* model), it performs better than the *HMAX* model in face/non-face classification tasks. Furthermore, we demonstrate that our proposed mechanism is capable of following similar trends in performance as humans in a psychophysical experiment using a face versus non-face rapid categorization task.

## Introduction

Although real-world object recognition is one of the most complex and difficult of tasks, it is robustly and rapidly performed by the primate visual system. The visual system can easily adapt itself to real-world object recognition, where objects are presented in cluttered backgrounds that can vary in illumination, viewpoint, position and scale. Neurobiological evidence demonstrates that object recognition in the visual cortex is mediated by the ventral visual pathway [1], which starts from the primary visual cortex *V1*, continues over the extrastriate visual areas, *V2* and *V4*, to the inferotemporal cortex (*IT*) and then to prefrontal cortex (*PFC*) [2]–[4]. This pathway exhibits a hierarchical structure in which the complexity of the preferred stimuli and the receptive field of cells correspondingly increase along the hierarchy [2], [3]. Based on widely accepted evidence, several models of visual cortex have been proposed. For example, a major breakthrough in this field has been derived from the work of Hubel and Wiesel on the cat [5], [6] and macaque primary visual cortex [7]. These studies demonstrate that the processing in the visual cortex follows a hierarchical structure. Following Hubel and Wiesel's pioneering proposal of a hierarchical model for the primary visual cortex, several hierarchical object recognition models have been developed. For example, Fukushima [8] proposed Neocognitron, a hierarchical multilayered neural network that is capable of robust visual pattern recognition through learning [9], [10]. Riesenhuber and Poggio [11] also proposed the *HMAX* model, which is based on the classical simple-to-complex cells model by Hubel & Wiesel. The *HMAX* model attempts to quantitatively resemble visual processing in the ventral visual pathway. A significant degree of invariance to scale and translation are some characteristic of the *HMAX* model. Furthermore, this model outperforms some state-of-the-art computer vision systems in applications such as object recognition and scene understanding [12].

Another group of models, including the *LAMINART* and *SMART* models, does not fall into the category of object recognition models. These models try to implement details of circuits and layers of the visual cortex. The *LAMINART* model [13]–[15] is a model of the visual cortex that attempts to implement details of layers and circuits in the lateral geniculate nucleus (*LGN*), and the *V1* and *V2* areas of the visual cortex. The Synchronous Matching *ART* model (*SMART*) [16] implements interactions between the laminar cortical circuits and higher-order thalamic nuclei. These models are based on the adaptive resonance theory, which was developed and inspired by how the brain performs information processing [17], [18].

Solving the stability-plasticity dilemma together with achieving memory stability in an evolving input environment is considered as a fundamental goal. The stability-plasticity dilemma is related to how our brain learns enormous amounts of information and can remain stable against forgetting previously learned material. The *LAMINART* and *SMART* models attempt to show how the *ART* mechanism may be embedded in the cerebral cortex and attempt to propose a solution to the stability-plasticity dilemma observed in the cerebral cortex.

Extracting biologically plausible visual features that can mimic visual processing in the primate brain has been a challenging goal for computational models of object recognition. For example, learning in the model proposed by Serre et al. involves a simple mechanism of selecting random patches from the training images [19]. However, random selection is not a biologically plausible approach. To select only relevant features for a given task, LeCun used a supervised back-propagation approach to learn visual features in a convolutional network [20]. M. Ghodrati et al. proposed a method which uses feedbacks from classifier (analogous to *PFC*) to extract informative visual features. Their method uses an optimization algorithm to select informative patches from a large pool of patches [21]. Masquelier et al. [22] used the spike timing-dependent plasticity (*STDP*) learning rule in an architecture on the basis of the Serre et al. model. Although this is a biologically-plausible approach, it is not stable due to the forgetting of previously learned information. Furthermore, each input is required to be presented several hundred times, whereas usually our brain is able to learn scenes at first glance.

In this paper, by using a stable visual feature learning mechanism, we propose a model which incorporates one of the well-know object recognition models (the *HMAX* model), that is based on the hierarchical model of Hubel and Wiesel. The *HMAX* model is a feedforward network of four layers of alternating simple and complex units (*S_1_, C_1_, S_2_, C_2_*). The *HMAX* model with our proposed feature learning mechanism, inspired by the *ART* system, suggests a mechanism for solving the problem of stability versus plasticity in object recognition systems. Both the *ART* mechanism, which is employed in our model, and the *STDP* rule are biologically plausible. However, the *ART* mechanism enables our model to learn informative features in a single presentation of the input image. This is in contrast to the *STDP* rule, which requires hundred times of image presentation.

There are some other object recognition models that have used the Adaptive Resonance Theory. For example, Woodbeck et al. [23] proposed a biologically plausible hierarchical structure which was an extension of the sparse localized features (SLF) suggested by Mutch et al. [24]. One of their contributions was that, instead of using support vector machines (SVM) for classification, they used *Fuzzy ARTMAP* as a biologically plausible multiclass classifier [25] which is based on the Adaptive Resonance Theory (ART). There are also some other studies that have employed Adaptive Resonance Theory to classify objects after extracting features [26], [27]. However, we have adopted Adaptive Resonance Theory for selecting informative visual features before classification stage in a learning mechanism. There are also many other pattern recognition systems based on the ART mechanism [28]–[32], which do not have a hierarchical structure inspired by the primate visual cortex.

We evaluated the proposed learning mechanism in a facial categorization task and compared the results with a benchmark model of object recognition; we also compared the performance of the both models with the performance obtained from a psychophysical experiment using human observers. Our results demonstrate that the proposed model has a higher classification performance than the benchmark model and resembles human responses at an acceptable level.

## Materials and Methods

### The stability-plasticity dilemma

Humans can memorize new faces at a glance, but this fast learning ability does not yield forgetting the previously known faces. The ability of our learning system to memorize novel events is called *plasticity*. In contrast, the ability that prevents the catastrophic forgetting of previously learned information is called *stability*. This mechanism, which exists in all adaptive processes of the brain, is called the stability-plasticity dilemma [18]. This dilemma hinges on the idea that human and mammalian brains are able to learn massive amounts of new information throughout their life without forgetting previously learned information.

**Figure 1:**
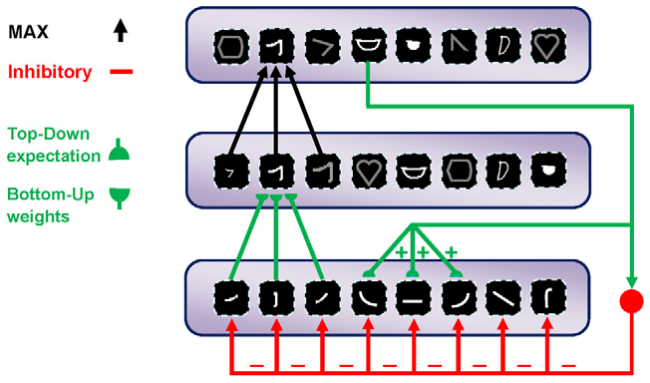
The top-down matching mechanism. The bottom-up weighted connections cause the activation of some units in the upper layer. These units send excitation signals to the relevant units through direct top-down weights and inhibit signals to all units and amplify the activities of cells within the matched (on-center) portion while suppressing the activities of irrelevant cells in the non-matched (surround) portion; thus, this network is named the on-center, off-surround network. The units in the first layer receive both excitation and inhibition (on-center), and additional excitations may overcome the inhibitions. In contrast, when the cells receive only top-down inhibition (off-surround), then one inhibition may counteract one excitation from the input.

One theory that addresses the stability-plasticity dilemma is the *ART*, which was proposed by Grossberg [17]. The *ART* is a cognitive and neural theory that attempts to provide a solution for the stability-plasticity dilemma. It proposes a top-down matching mechanism in which bottom-up signals activate top-down expectations; this attracts attention to the relevant information in the bottom-up pathway (Figure 1). The *ART* works with an on-center, off-surround network that amplifies the activities of the cells within the matched portion (on-center) while suppresses the activities of irrelevant cells in the non-matched portion (the surround) (Figure 1). The top-down modulatory on-center, off-surround circuit [33]–[37] is used for the matching process in our proposed model. We used this matching process for selecting attended features and inhibiting unattended ones. This proposed model makes use of the bottom-up adaptive weights as well as the top-down expectations, which enables the attended feature patterns to be learned. If the input pattern adequately matches the top-down expectations, then these top-down expectations will reactivate relevant bottom-up pathways, thereby generating a state of feedback resonance between the bottom-up and top-down pathways. In contrast, a large mismatch can lead to hypothesis testing or searching for a new and more predictive category.

**Figure 2:**
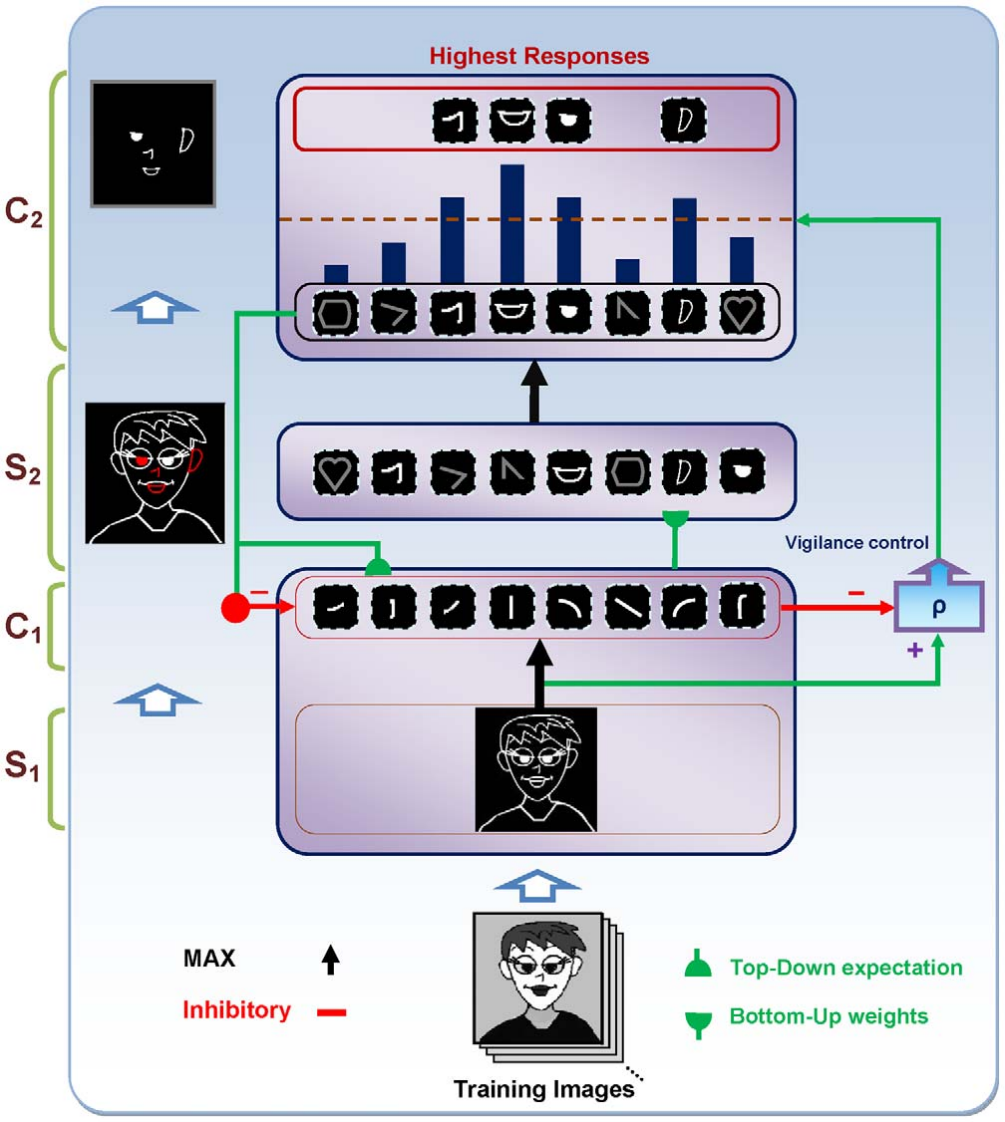
A schematic diagram of the proposed model architecture. Grayscale images are applied to the system and the outputs of *S_1_* and then *C_1_* are attained. Then, the *S_2_* responses are computed using existing prototypes. Next, to compute the *C_2_* responses, the *S_2_* units with the maximum response for each prototype for all positions and scale bands are selected. The highest active *C_2_* units are then selected as prototypes to represent the image (these are shown in the red box at the top of the figure). This selection is achieved by top-down expectations, which match the input image to the prototypes. A lateral subsystem (vigilance control), which uses a vigilance parameter (*ρ*), determines the matching degree between the prototypes and various parts of the input image. If a selected active *C_2_* unit has a smaller response than the vigilance value, then a new prototype is extracted from the current input image and added to the existing prototypes.

As previously described, top-down connections exist in the early layers of the visual cortex such as *V1* and *V2*, which demonstrates that the visual cortex not only has feed-forward connections (unlike the classical model of Hubel and Wiesel), but also possesses feedback connections, which is thought to have a key role in the stabilization of both development and learning within multiple cortical areas including the *V1* and *V2* areas [40]. Therefore, the feedback loop from complex cells to simple cells through a modulatory on-center, off-surround network can be thought of as an implementation of *ART* matching in the visual cortex.

### The stability-plasticity dilemma in the visual cortex

How the visual cortex automatically develops circuits and can still remain stable is a major question for which several models have been developed, including the *LAMINART* model [13], that attempts to implement details of the layers and circuits of the *LGN*, *V1,* and *V2* areas in the visual cortex. The Synchronous Matching *ART* model [16] is another example, which goes beyond the *LAMINART* model and implements interactions between laminar cortical circuits and higher-order thalamic nuclei. The *LAMINART* and *SMART* models are based on the adaptive resonance theory, which suggests a solution for the stability-plasticity dilemma.

Simple cells in the *V1* area receive direct inputs from the *LGN* and also from an on-center, off-surround network [38]. Complex cells receive inputs from simple cells with the same orientation but different contrast polarities and can thus respond to both polarities. In addition to these bottom-up connections, cortical connections of the visual cortex have been shown to provide feedback to lower level layers. For instance, active complex cells send top-down signals to simple cells through an on-center, off-surround network, and simple cells in turn activate complex cells. This feedback process is called folded feedback (see Figure 2B in [14]). The top-down signals from complex cells to simple cells, using an on-center off-surround network, enables highly active complex cells to inhibit lower active cells [39]. The *V2* circuitry also demonstrates a similar pattern to that of *V1*, but on a larger spatial scale.

### The proposed model

We propose a biologically motivated object recognition model which incorporates the *HMAX* model, and uses a stable learning method, inspired by the *ART* mechanism, to solve the stability-plasticity dilemma. The proposed model is generally based on *Neocognitron* [9] and *HMAX* (which is another hierarchical model based on *Neocognitron*) proposed by Riesenhuber and Poggio [11]. Some parameters of the model proposed in this study, particularly those in the edge detection stage, have been adjusted to be comparable with the *HMAX* model in facial categorization tasks (We used the HMAX MATLAB implementation, which was freely available at http://cbcl.mit.edu/software-datasets/index.html). Furthermore, to solve the stability-plasticity dilemma, we used the *ART* mechanism to extract more informative features of intermediate complexity, and this consequently provides a more realistic biologically inspired model.

**Figure 3:**
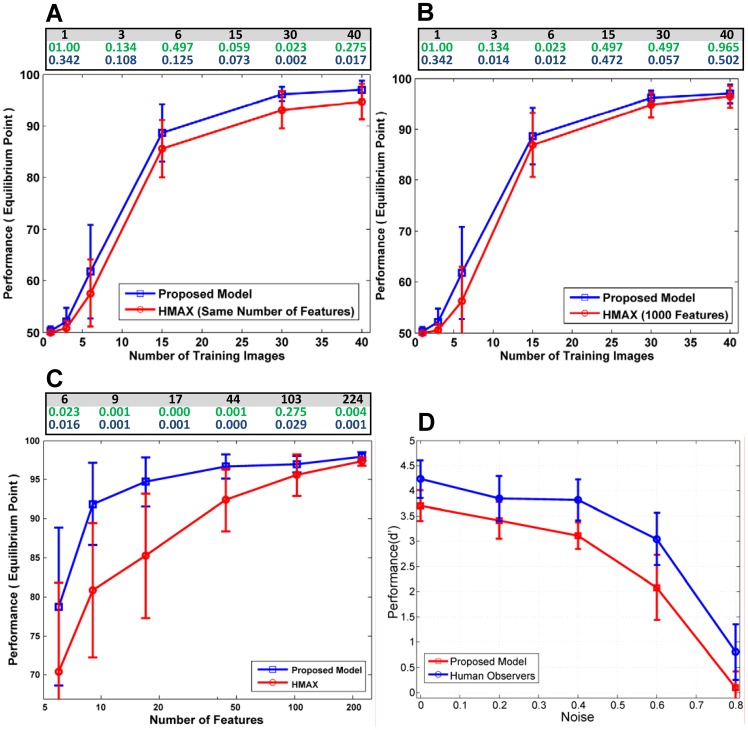
Various comparisons between the proposed model, another biologically plausible model and human subjects. (A, B), The performance achieved across different number of training images. (C), The performance achieved across different number of features (green digits are p-values obtained using the Wilcoxon-rank sum test [49] and dark blue digits are those obtained from the two-Sample Kolmogorov-Smirnov test [50]). (D), The average performance achieved by the human observers, the proposed model, and the *HMAX* model on images with various levels of noise, error bars are standard deviation (SD).

The proposed model has a hierarchical structure and intends to emulate rapid object categorization in the visual cortex. The model consists of alternating simple and complex units: simple (*S*) units correspond to the simple cells in the visual cortex, which combine their inputs according to a bell-shaped tuning function to increase selectivity. Complex (*C*) units correspond to the complex cells in the visual cortex, which show tolerance to a shift in the position and size of the stimuli within their receptive field. These units pool their inputs through a maximum (max) operation [11] to increase invariance (biologically plausible circuits for these two operations can be found in [41]). The proposed model consists of four layers of alternating simple and complex units (Figure 2). The *S_1_* units take the form of the Gabor function [42] and convolve the input image to detect bars and edges. The Gabor function has many free parameters, which agrees well with physiological data recorded from simple cell receptive fields in cat striate cortex [43]. The parameters of the Gabor function were set up to match the tuning properties of simple cells in *V1*. The *S_1_* units include 16 filter sizes, spanning a range of sizes from 7×7 to 37×37 pixels in steps of two pixels, and four orientations (0°, 45°, 90°, 135°). Totally, there are 64 different *S_1_* units. These 64 filters are then divided into eight bands where each band contains two adjacent filter sizes [12].

Each of the complex *C_1_* units pools its inputs over a group of simple *S_1_* units which have the same preferred orientation but at slightly different positions and sizes. The index of the filter size bands determines the pool range for the *C_1_* units. This pooling increases the invariance to the changes in shift and size inside the receptive field of the units.

**Figure 4:**
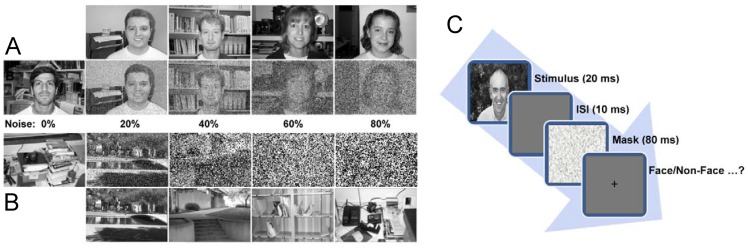
Generated images with different levels of noise. (A), Examples of faces. The first row consists of noise-free images, and each noisy image in the second row corresponds to the above noise-free image. (B), Examples of distractors. The first row consists of noisy images, and the second row corresponds to noise-free images. (C), The psychophysical task process. A face image is presented for 20 ms, and then a blank screen is presented (*ISI* 10 *ms*). Next, a noisy mask is presented for 80 *ms*. Finally, the subject is asked to select “YES” or “NO” by pressing the appropriate key on a computer keyboard.

The next layer is *S_2_*, which is selective to more complex patterns than bars or edges within their receptive field. The units of this layer receive their input from retinotopically organized *C_1_* units in a spatial grid and in all four orientations via weighted connections that respond to specific patterns or prototypes, bottom-up weights (Figure 1).

The last layer of the model consists of *C_2_* units that respond to the prototypes of the input image extracted from different locations, which increases invariance. A *C_2_* unit has connections with *S_2_* units of the same prototype but in a different size and position. Thus, the results of this layer are *C_2_* values in a vector of size *N*, where *N* is the number of prototypes learned by the model. The *C_2_* responses illustrate the matching between the prototypes and the input image. A high *C_2_* response indicates that the extracted prototype is sufficiently matched by a portion of the input image and is thus suitable for representing the input image.

The feedback from complex cells to simple cells through the on-center, off-surround network in the *V1* and *V2* areas of the visual cortex leads to the excitation of related simple cells by winner complex cells and inhibits irrelevant cells. In addition to the feedback from complex cells to simple cells, the feed-forward connections between simple and complex cells create a feedback loop that yields a resonant state for relevant cells [39].

**Figure 5:**
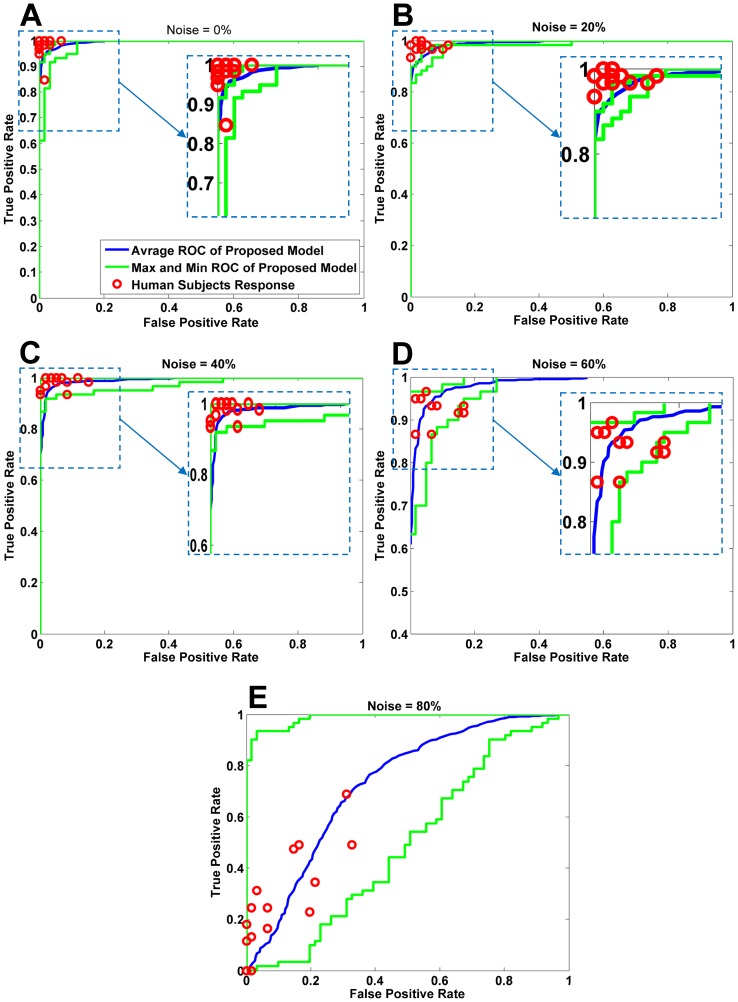
ROC for the proposed model and humans. The upper and lower green curves correspond to the maximum and minimum ROC curves for the proposed model, and each of the red circles corresponds to the results obtained by a human observer. The average of these ROC curves is shown by the blue curve.

According to this feedback loop, we simulate this match learning to learn informative intermediate-level visual features from the input images. This feedback excites portions of inputs that are matched by the prototypes of the active *C_2_* units and inhibits portions of inputs that are not matched by these prototypes (Figure 1). In contrast, if the mismatch is higher than the value of vigilance parameter (this parameter is explained later), this means that the existing *C_2_* units are unable to represent the input image. Next, new prototypes from the current input are extracted and added to the preceding *C_2_* units. In other words, we assume that for each input image, *P* numbers of *C_2_* units are sufficient to represent the image. If these *P* features were previously available in the current pool of patches, we would have an accurate representation of the input image. Otherwise, the new patches will be extracted and added to the pool of patches. To achieve informative prototypes for each image, we employed the match learning and reset mechanism of the *ART* system (Figure 2). An analogy can be seen between adding new *C_2_* units and match-based learning, which has been suggested to be a learning mechanism in the brain. Match-based learning updates memory only when a completely new input occurs or there are some inputs from the external world, which are sufficiently close to internal expectations [16].

We presented all of the training images to the system, and outputs of *S_1_* and then *C_1_* were attained. The *S_2_* responses were then computed by utilizing the existing prototypes. Next, to compute the *C_2_* responses, the *S_2_* units with a maximum response for each prototype for all of the positions and scale bands were selected. We selected *P C_2_* units with the highest activity to represent the image (this selection was achieved by top-down expectations, which match the input image to prototypes) and compared them with a vigilance parameter to determine the matching degree between the prototypes and the input image. These selected units are shown separately at the *C_2_* level (Figure 2). If the amount of matching is lower than the vigilance, then the prototype will not represent the input image appropriately and results in extracting new prototypes from the current image and adding them to the prototype pool. Using this learning process, with a single presentation of an image of the training set, proper prototypes that represent the image are efficiently extracted.

**Figure 6:**
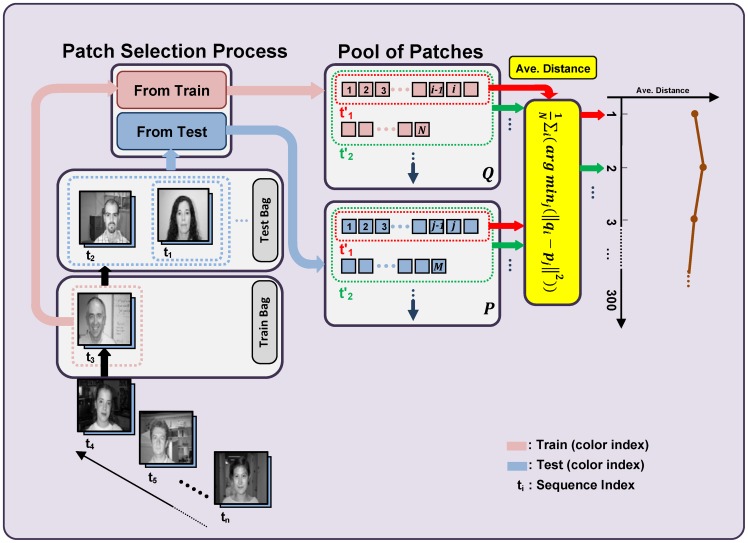
Details of the process used to compare the stability of the proposed model with the *HMAX* model. The first iteration can be explained as follows: first, two images (i.e., the t_1_ set) are randomly selected and sent to the train bag. Next, both the proposed model and the *HMAX* model are used to extract a pool of patches called t_1_ patches, which are depicted in the red-colored dashed box (box Q). Subsequently, patches are extracted of two other randomly selected images (i.e., the t_2_ set), and these patches are added to the pool of training patches, indicated by the pink-colored squares in box Q. The process is continued by extracting patches of all of the images in the test bag and storing them in box P (the patches are shown as pale blue-colored squares). Next, the average distances between all the training and test patches, which are shown in boxes Q and P, respectively, are computed.

To control the generality of the learned features, a vigilance parameter in the model was used that is analogous to the process mediated by acetylcholine. According to the *SMART* model [16], a combination of nonspecific nuclei and the nucleus basalis of Meynert is proposed to play the role of the vigilance parameter in our model (see Table 1 in [16]). The vigilance parameter is set in such a way to attain the highest performance with the fewest prototypes. The selection of the vigilance parameter is highly critical in an *ART* network, and there is no special rule for setting the value of vigilance [44]. To determine the vigilance parameter, a group of images were randomly selected from the dataset prior to the training and testing stages. Next, from these images, the vigilance parameter was specified manually. Finally, the vigilance parameter remained fixed during both the training and testing stages for these experiments.

*The classification stage*: to compare our model with the *HMAX* model [12] in a face/non-face categorization task, we added a classification stage to the model that is similar to that of the *HMAX* model. For all images in the training and testing sets, each image was passed through the layers of the model, and the responses of the *C_2_* units were computed and saved as a vector representing the extracted features for that image. Next, these vectors were subsequently passed to a linear classifier (Simple linear *SVM* classifier) for classification.

**Figure 7:**
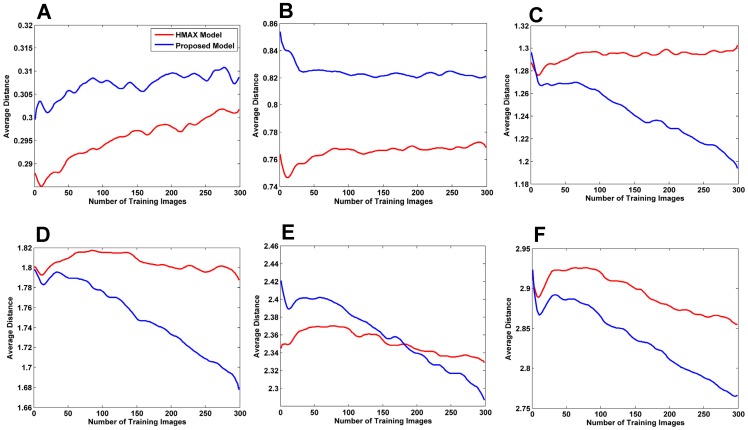
Investigating the stability of the different patch sizes . (A–F) show the average minimum distances between the test and training patches for the six patches of sizes 4, 8, 12, 16, 20, and 24. As the trend moves downward, it indicates that more diverse prototypes are being learned and that these prototypes are able to better represent the test images. (A), The result for patch size 4; the proposed model shows an upward trend, and thus, this specific patch size is not stable along the image presentation sequence. (B–F), As can be seen, the proposed model exhibits steeper downward slopes in most cases unlike the *HMAX* model, which mostly shows upward trends. In E and F, both models exhibit downward trends; however, the trend in the proposed model demonstrates a steeper downward slope.

### Images dataset

To evaluate the performance of the proposed model, we used the face image category of the widely used California Institute of Technology (Caltech101) datasets [45]. These datasets consist of 101 different object classes as target images and a background folder as negative examples. We used the background dataset as distractor images. The face dataset contains face images of various people against various backgrounds in various positions. This dataset appears to be challenging for facial categorization. The number of images in the face and background datasets are 435 and 451, respectively. The dataset is freely available at http://www.vision.caltech.edu/Image_Data sets/Caltech101 (This dataset is completely free and has been widely used and represented by authors. Some researchers who have used these face images in their work include [22], [45]–[48]).

**Figure 8:**
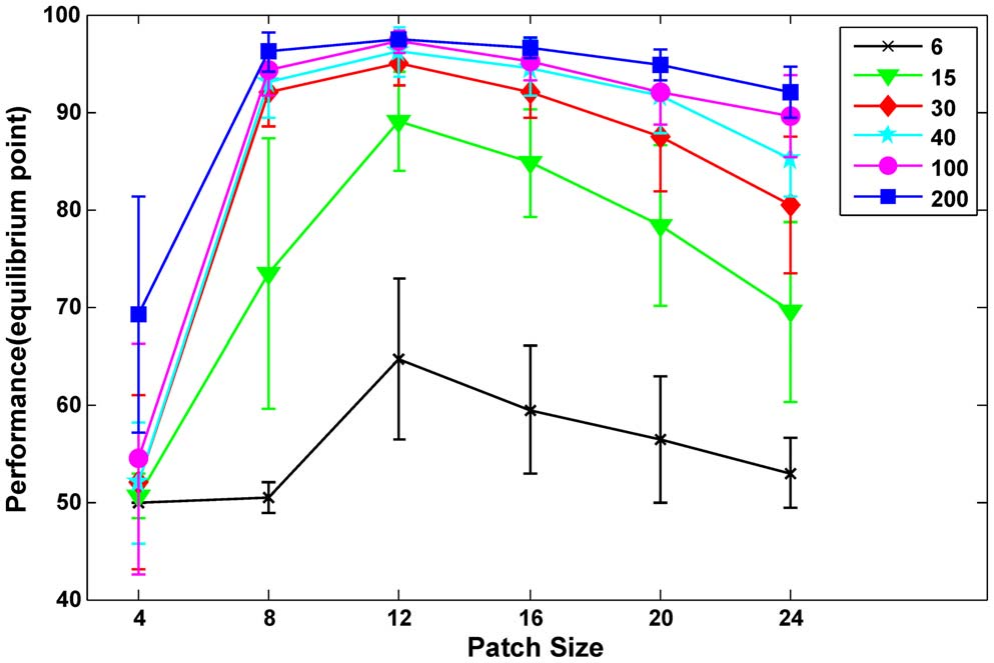
Performance for all patch sizes. Horizontal axis shows six different patch sizes (from 4 to 24) and vertical axis reports the performances for each patch size with different number of positive training images, each colored line illustrates an specific number of training image (specified with black for six images to blue for 200 images).

### Classification by the Proposed Model

We designed various experiments to compare the proposed model with the *HMAX* model in face/non-face categorization tasks. The images were converted to grayscale values and rescaled to be 140 pixels in height. The width was rescaled accordingly to preserve the aspect ratio. In all experiments, the following procedure was performed:

1. *Extracting C_2_-level features:* Our stable fast learning algorithm was performed on the training dataset to extract a set of *C_2_*-level features.
2. *Training the SVM classifier:* All of the training set images were applied one by one to the model, and *C_2_* responses were calculated. The *C_2_* responses with labels (1 for positive and -1 for negative examples) were used to train a classifier (i.e., the Simple linear SVM classifier). It is noteworthy that layers are fixed at this stage, and learning in lower levels of the system is stopped.
3. *Evaluating the extracted features:* The performance of the classifier on the test set was evaluated. The overall procedure was repeated 20 times, and the average performance and standard deviation (SD) were reported.

In the first experiment, we evaluated the performance of the proposed model in a face/non-face classification task. For this purpose, the datasets were randomly divided into two subsets with equal number of images, i.e., for the training and test sets. The first subset was used for extracting *C_2_*-level features and training the *SVM* classifier, and the second subset was used for evaluating the classification performance.

In the next experiment, we studied the effect of the number of training samples on the classification performance. The model was evaluated using different numbers of positive training samples (1, 3, 6, 15, 30, and 40). We used 50 negative training samples, 50 positive test samples, and 50 negative test samples. To demonstrate that our model extracts informative and as few intermediate-level features as possible from the images, we measured the classification performance across different number of extracted features.

For further studies regarding the biologically plausibility of the proposed model, we compared the performance of the face/non-face categorization task in humans with the model.

## Results

In the next two sections, we report the results of different comparisons made between the proposed model, another biologically plausible model (*HMAX*), and human subjects. First, the results of the proposed model are compared against the *HMAX* model in three different experiments. As a follow-up to these results, we compare the performance of the human subjects in a psychophysical test (rapid categorization of faces versus non-faces) with the performance of our proposed model.

### Comparison with another biologically plausible model

We compared our results against another established biologically motivated object recognition model, the *HMAX* model. This model outperformed many machine-vision object recognition systems at several tasks [12]. We evaluated the performance of the *HAMX* model using the proposed visual feature learning mechanism against the standard *HMAX* model. For this purpose, we used the face category of Caltech101.

In the first experiment, the face and background datasets were randomly divided into two separate sets of equal sizes. Next, we applied our stable fast-learning algorithm to the training dataset to extract the most informative intermediate-level features from the images. The vigilance parameter in the model was determined such that the most informative features with the highest possible performance were extracted. After this stage, the prototype learning was stopped, and the obtained features were applied in the face/non-face classification task. The classification performance of these features was then computed. In the classification stage, we used a linear *SVM* classifier. The performances were reported with an accuracy measure at the equilibrium point, which occurs at the accuracy point when the false positive rate equals the missed rate. For a fair comparison, we also used the *HMAX* model on the same training and test set. The classification performance was 98.5% for our model and 98% for the *HMAX* model. To determine whether the performance differences between the *HMAX* model and the proposed model were statistically significant, we used two non-parametric statistical tests, i.e., the Wilcoxon rank sum [49] and the two-Sample Kolmogorov-Smirnov test [50] (Implemented in MATLAB statistical toolbox. Under the null hypothesis the distribution and mean of both groups are equal, so that the probability of an observation from one population (X) exceeding an observation from the second population (Y) equals the probability of an observation from Y exceeding an observation from X. Note that, distributions are classification performances obtained over 20 independent runs of the *HMAX* model with random feature extraction, and with the proposed feature learning mechanism. Under the alternative hypothesis, the probability of an observation from one population (X) exceeding an observation from the second population (Y) is not equal to 0.05. Rejection of the null hypothesis is at the 0.05 significance level. The reported p-values using these methods were 0.009 and 0.059, respectively.

To study the potential effects of using a different number of training examples on the performance of the system, we selected 1, 3, 6, 15, 30, and 40 positive training samples. We used 50 negative training samples together with 50 positive and 50 negative test samples. The performances of the proposed model and the benchmark model using different number of training images are compared in Figure 3A (experiments were independently performed 20 times. Afterwards, the average performance and standard deviation were reported). For each training stage run and after giving the training data to the proposed model, sufficient informative *C_2_*-level-features were extracted and subsequently used to train the classifier for the face/non-face classification task. We then implemented the benchmark model using the same number of features. To make the comparison more challenging for our proposed model, we also performed the benchmark model using 1,000 features. As shown in Figure 3A and 3B, the performance of the proposed model was better than the benchmark model (for the same number of features) across different number of training examples; the proposed model also performed moderately better than the benchmark model with 1,000 features. In some cases in Figure 3A and 3B our results are not statistically significant. However, when we use fewer features, as it can be seen in Figure 3C, the classification performances of our method are significantly better, and p-values reveal that the results in this case are statistically significant.

In the next experiment, we compared the performance of the proposed model with the *HMAX* model using different numbers of features. In this case, the parameters of the proposed model were set to extract a number of features, and these features were then used in the classification task. The datasets were randomly divided into two separate subsets of equal size (the training set and the test set). As shown in Figure 3C, when we use fewer numbers of features, our proposed model significantly outperforms the benchmark model. For example, the proposed model had significantly better performance when using fewer features (e.g., approximately 93% with only 9 features) than the benchmark model (e.g., approximately 80% with 9 features). This demonstrates that the proposed visual feature learning mechanism can strongly improve the performance of the *HAMX* model using very few features in contrast to the *HMAX* model with randomly extracted features. This finding illustrates that our mechanism has extracted more informative features from the input images than did the standard *HMAX* model. With such a biologically plausible learning mechanism, we addressed the stability-plasticity dilemma and also solved the problem of extracting redundant features.

Moreover, the proposed model suggests a stable biologically plausible learning mechanism for extracting intermediate level visual features (for more information regarding the stability of the proposed model please refer to the stability of the proposed model section).

### Comparison with human

We performed a psychophysical experiment for categorizing faces versus non-faces to compare the proposed model with human observers. For this purpose, we selected the Caltech face and background datasets as positive samples and distractors, respectively. In addition, we added various levels of salt and pepper noise to these images. The noisy images were shown to human subjects using a computer screen in a random order. The human subjects were instructed to respond as fast and as accurately as possible to determine whether the image contains a human face or a distractor by pressing the ‘‘YES’’ or ‘‘NO’’ key. The results obtained from the human subjects were compared with those obtained using the proposed model on the same image set.

We used 16 human subjects in this experiment (18–36 years old) with an equal number of male and female subjects. The Stimulus Onset Asynchrony (*SOA*) in this test was a fixed *SOA* of 30 *ms* (20 *ms* image presentation followed by an Interstimulus Interval (*ISI*) of 10 *ms*). The experiment was performed in a dark room. The participants were seated 0.5 *m* away from the computer screen (Intel core 2 duo processor (2.66 *GHz*), 4 GB RAM). We used the MATLAB software with the psychophysics toolbox [51]–[53]. In the experiment, the image was presented for 20 *ms*, and this was followed by the presentation of a random noise mask. The mask appeared after a fixed *ISI* for duration of 80 *ms* (which corresponded to an *SOA* of 30 *ms*). Please refer to Figure 4C for additional details of the psychophysical experiment procedure.

To pose a variety of challenges to the task, we used five sets consisting of an equal number of images in each set (60 faces and 60 distractors at the same level of noise in each set, 600 stimuli in total). These five sets correspond to various levels of noise (0, 20, 40, 60, and 80 %; see Figure 4). These images (300 faces and 300 distractors) were randomly selected from both the face and background datasets. Next, various levels of salt and pepper noise were generated and superimposed on the images in each group. The images were presented in a random order at the center of the screen (256*300 pixels, grayscale images). Each image only appeared once to omit the potential for image-specific learning effects. The subjects were then asked to accurately respond as fast as they could as to whether the image contained a human face or a distractor image by pressing the ‘‘YES’’ or ‘‘NO’’ key on the computer keyboard. In addition, the subjects were alternately asked to use their left or right hand to press the ‘‘YES’’ vs. ‘‘NO’’ key. Each experiment lasted approximately 15 minutes. The remaining images for both the face and distractor datasets were used to extract *C_2_*-level features and to train the classifier in the proposed model. Obviously, the training images were noise-free. Next, we evaluated the performance of the classifier on the “test” set.

A comparison between the average performance of the human observers (n = 16, 30 *ms SOA*) and the proposed model in the face/non-face classification task is shown in Figure 3D. The performance was measured using a performance measure *d*′, which combines both the hit and false-alarm rates of each observer into a single standardized score. The responses of both the proposed model and the human subjects were roughly similar. The proposed model was capable of following similar trends in responses as humans in this experiment. The performance of the *HMAX* model for this experiment is also demonstrated in Figure 3D (green line).

We also compared our results with human responses using *ROC* curves. The blue curve in Figure 5 was obtained by averaging all of the *ROC* curves across 10 random runs and the upper and lower green curves are the maximum and minimum *ROC* curves, respectively, which correspond to the highest and lowest performance of the proposed model in the different runs. Because it is impossible to use the *ROC* curve for the human observer responses, we represented the true positive to false positive ratio of each subject using the sixteen red circles shown in Figure 5 (we magnified some important parts of the plots in Figure 5 for better visualization). The majority of the red circles were located below the maximum, above the minimum, and adjacent to the average *ROC* curves, which implies that the proposed model nearly resembles the performance of the human observers.

### Stability of the proposed model

One interesting property of the proposed model is its stability, which means that after learning new features, the model is still capable of remembering previously learned ones. To examine the stability of the model, we designed an experiment that enabled the measurement of the stability of the proposed model and the comparison of its stability with the *HMAX* model.

For the purpose of measuring stability, we trained each model using *n* images, and subsequently, added *m* new images to both trained models. The two models were compared to determine how well the different models retained the first *n* trained images. For each iteration of this procedure, while previously learned features are preserved, we present *m* new training images to both models. In this step, our stable visual feature learning mechanism will only extract new patches in which the vigilance parameter determines whether the patches are necessary to be added to the previously learned pool of patches; in this way, the new pool becomes more capable of representing these new *m* images. However, in the *HMAX* model, the same number of patches is randomly extracted. Next, in the test phase, we extract new patches from all of the preceding images except for the recent *m* training images. Then the average of the minimum distance between these two groups of patches is computed (for details see Figure 6) to determine how similar the extracted patches remain to the previously extracted patches after adding *m* new training images. We consider this average distance a measure for comparing the stability of the models (additional details are depicted in Figure 6). In each step, we present two new images, and new training patches are extracted from these new images.

Figure 7 provides information about the stability of our proposed approach, which was trained with various patch sizes. In this experiment, we compared the stability of the proposed model with that of the *HMAX* model. The average minimum distances between the test and training patches for six patches of sizes 4, 8, 12, 16, 20, and 24 are reported. In general, a downward trend in each curve indicates that the average distance decreases on adding new training images, thus confirming that the model will not forget previously learned features (as the slop goes steeper, it shows more stability). For more clarification, imagine the model has learned some features from an input image, then, by adding a new image to the model, it may require learning new features or may not. Therefore, the model learns new features only when the previously learned features are insufficient for describing the new input image. As a result, the average minimum distance decreases in each stage because the model does not forget previously learned features and only extracts new required features. If the model was not stable, it would extract non-required features in every stage which would result in an increase to the average minimum distance. As observed, the proposed model exhibited steeper downward slopes in most cases unlike the *HMAX* model, which mostly shows upward trends. In Figure 7E and 7F, both models exhibit downward trends; however, the trend in the proposed model has a steeper downward slope. With the exception of patch size 4 (Figure 7A), for which the proposed model showed an upward trend, indicating that this specific patch size was not stable. This could be due to the small area that a patch of size 4 covers. This small patch size may not cover important discriminative components of the face in an image; therefore, it may not able to separate a face from a distractor sufficiently well (Figure 8 also illustrates that the performance was close to chance level for patch size 4).

We also probed the relationship between the performance and stability of the model by running an experiment for all of the patch sizes separately using a different number of training images. In this experiment, the classification performance was measured for each patch size. As shown in Figures 7 and 8, we observed that when the proposed model is trained with patches that are more stable, better performance could be obtained. This suggests a direct relationship between the stability of the proposed model and its performance.

## Discussion

The most widely accepted biological evidence shows that visual processing in the brain exhibits a hierarchical structure, which starts from the primary visual cortex (*V1*), and then continues to the extrastriate visual areas (*V2* and *V4*), which are next followed by the inferotemporal cortex (*IT*) and the prefrontal cortex (*PFC*).

It is thought that plasticity and learning probably occurs at all stages, in particular, at the level of the *IT* and *PFC* [54]. The way by which this learning and plasticity occurs in the cortex has been a major concern in computational models of the visual cortex. For example, the learning process in the proposed model of Serre and colleagues occurs only between layers *C_1_* and *S_2_* which is a simple mechanism of indiscriminately selecting patches from the training images [19]. This approach leads to acceptable results, but redundancy between features is very high. Moreover, many of the features may be irrelevant to the task of classification. This increases the cost of classification and decreases the performance. However, random selection is not a biologically plausible approach. Apart from random selection, some other approaches have been suggested including the use of a supervised back-propagation approach to learn the visual features in a convolutional network. Another potential approach used the *STDP* learning rule to extract intermediate-complexity visual features [22]. These features have been shown to exhibit robust object recognition in some classification tasks. However, due to the nature of the *STDP* rule, which causes forgetting previously learned information, this approach is unstable. Furthermore, for the sake of learning by this rule, each input must be presented several hundred times, whereas our brain is able to learn scenes at a glance. In contrast to the *STDP* rule, we proposed another approach for the learning of intermediate-level features, which is not only a biologically plausible method but also addresses the problems of instability, the need for repeated image presentation, and the issue of the redundancy of the extracted visual features in the *HMAX* model. Whereas other models do not illustrate how the visual cortex is stable against the destruction of previously learned information over time, our model applied the *ART* mechanism, which solves the stability-plasticity dilemma. We showed that the proposed model is capable of learning new information without losing previously learned information. We also demonstrated that there is a direct relationship between the stability of the model and its performance. This means that if the model is trained with more stable patches, it performs better. This mechanism was implemented in a hierarchical feed-forward model of the visual cortex and used in face categorization. We also compared our results with the *HMAX* model in face/non-face categorization tasks, and the obtained results showed that it performed better than the *HMAX* model in ‘different number of training images’ experiment although not significant. However, our model significantly outperformed the *HMAX* model in ‘different numbers of features’ experiment, particularly with fewer numbers of features. Performed experiments using different numbers of features showed that our model extracts as fewest as possible features from the training images, which are the most informative features; and yet achieves an acceptable performance. In contrast, the *HMAX* model requires extracting more features to reach the similar performance. This showed that features learned by the proposed mechanism are highly informative which makes them capable of giving much better representation of the input images in higher processing layers. This thus results in improving the classification performance while using fewer numbers of features, as shown in Figure 3C.

To determine to what extent the proposed model can mimic the performance of human subjects, we performed the same face/non-face categorization task on humans in a rapid categorization psychophysical test. Our results showed a trend using the model that approximately resembles the trend observed in human subjects.

## References

[1] Ungerleider LG, Haxby JV (1994). “What” and “where” in the human brain.. Curr Opin Neurobiol.

[2] Perrett DI, Oram MW (1993). Neurophysiology of shape processing.. Image Vision Comput.

[3] Kobatake E, Tanaka K (1994). Neuronal selectivities to complex object features in the ventral visual pathway of the macaque cerebral cortex.. Journal of Neurophysiology.

[4] Tanaka K (1996). Inferotemporal cortex and object vision.. Annual review of neuroscience.

[5] Hubel DH, Wiesel TN (1962). Receptive fields, binocular interaction and functional architecture in the cat's visual cortex.. J Physiol.

[6] Hubel DH, Wiesel TN (1965). Receptive fields and functional architecture in two nonstriate visual areas (18 and 19) of the cat.. Journal of Neurophysiology; Journal of Neurophysiology.

[7] Hubel DH, Wiesel TN (1968). Receptive fields and functional architecture of monkey striate cortex.. J Physiol.

[8] Fukushima K (1980). Neocognitron: A self-organizing neural network model for a mechanism of pattern recognition unaffected by shift in position.. Biol Cybern.

[9] Fukushima K (1988). Neocognitron: A hierarchical neural network capable of visual pattern recognition.. Neural networks.

[10] Fukushima K (2003). Neocognitron for handwritten digit recognition.. Neurocomputing.

[11] Riesenhuber M, Poggio T (1999). Hierarchical models of object recognition in cortex.. Nat Neurosci.

[12] Serre T, Wolf L, Bileschi S, Riesenhuber M, Poggio T (2007). Robust object recognition with cortex-like mechanisms.. IEEE transactions on pattern analysis and machine intelligence.

[13] Grossberg S, Mingolla E, Ross WD (1997). Visual brain and visual perception: How does the cortex do perceptual grouping?. Trends in neurosciences.

[14] Grossberg S (2003). How does the cerebral cortex work? Development, learning, attention, and 3-D vision by laminar circuits of visual cortex.. Behav Cognit Neurosci Rev.

[15] Grossberg S (2007). Towards a unified theory of neocortex: laminar cortical circuits for vision and cognition.. Progress in Brain Research.

[16] Grossberg S, Versace M (2008). Spikes, synchrony, and attentive learning by laminar thalamocortical circuits.. Brain Res.

[17] Grossberg S (1976). Adaptive pattern classification and universal recoding: II. Feedback, expectation, olfaction, illusions.. Biological cybernetics.

[18] Grossberg S (1980). How does a brain build a cognitive code?. Psychological review.

[19] Serre T, Wolf L, Poggio T (2005). Object recognition with features inspired by visual cortex Ieee, Vol. 2.. 994–1000 vol.

[20] LeCun Y, Bengio Y (2003). Convolutional networks for images, speech, and time series. The Handbook of Brain Theory and Neural Networks. Cambridge, MA: MIT Press.. p.

[21] Ghodrati M, Khaligh-Razavi SM, Ebrahimpour R, Rajaei K, Pooyan M (2012). How Can Selection of Biologically Inspired Features Improve the Performance of a Robust Object Recognition Model?. PloS one.

[22] Masquelier T, Thorpe SJ (2007). Unsupervised learning of visual features through spike timing dependent plasticity.. Plos Comp Biol.

[23] Woodbeck K, Roth G, Chen H (2008). Visual cortex on the GPU: Biologically inspired classifier and feature descriptor for rapid recognition. Computer Vision and Pattern Recognition Workshops, 2008. CVPRW'08.. IEEE Computer Society Conference on.

[24] Mutch J, Lowe DG (2008). Object class recognition and localization using sparse features with limited receptive fields.. International Journal of Computer Vision.

[25] Carpenter GA, Grossberg S, Markuzon N, Reynolds JH, Rosen DB (1992). Fuzzy ARTMAP: A neural network architecture for incremental supervised learning of analog multidimensional maps.. Neural Networks, IEEE Transactions on.

[26] Kadiran S, Patnaik LM (1993). Distortion-invariant object recognition using adaptive resonance theory. Artificial Neural Networks and Expert Systems, 1993.. Proceedings., First New Zealand International Two-Stream Conference on.

[27] Uysal M, Akbas E, Yarman-Vural FT (2006). A hierarchical classification system based on adaptive resonance theory.. Image Processing, 2006 IEEE International Conference on.

[28] Zikan K, Caudell TP (1991). D-ART: a pattern recognition system based on adaptive resonance and algebraic metric space theories. Neural Networks, 1991., IJCNN-91-Seattle International Joint Conference on. Vol. 2.. p.

[29] Liao IE, Shieh SL, Chen HC (2008). An Evolutionary Classifier Based on Adaptive Resonance Theory Network II and Genetic Algorithm. Intelligent Systems Design and Applications, 2008. ISDA'08. Eighth International Conference on. Vol..

[30] Akhbardeh A, Varri A (2005). Novel supervised fuzzy adaptive resonance theory (SF-ART) neural network for pattern recognition.. Intelligent Signal Processing, 2005 IEEE International Workshop on.

[31] Carpenter GA, Martens S, Mingolla E, Ogas OJ, Sai C (2004). Biologically inspired approaches to automated feature extraction and target recognition. Applied Imagery Pattern Recognition Workshop, 2004. Proceedings..

[32] Antón-Rodríguez M, Díaz-Pernas FJ, Díez-Higuera JF, Martínez-Zarzuela M, González-Ortega D (2009). Recognition of coloured and textured images through a multi-scale neural architecture with orientational filtering and chromatic diffusion.. Neurocomputing.

[33] Grossberg S, Stone G (1986). Neural dynamics of word recognition and recall: Attentional priming, learning, and resonance.. Psychological Review.

[34] Carpenter GA, Grossberg S (1987). A massively parallel architecture for a self-organizing neural pattern recognition machine.. Computer vision, graphics, and image processing.

[35] Carpenter GA, Grossberg S (1991). Pattern recognition by self-organizing neural networks.. The MIT Press.

[36] Grossberg S (1995). The attentive brain.. American Scientist.

[37] Grossberg S (1999). How does the cerebral cortex work? Learning, attention, and grouping by the laminar circuits of visual cortex.. Spatial Vision.

[38] Ferster D, Lindström S (1983). An intracellular analysis of geniculo-cortical connectivity in area 17 of the cat.. J Physiol.

[39] Bullier J, Hupé JM, James AC, Girard P (2001). The role of feedback connections in shaping the responses of visual cortical neurons.. Progress in Brain Research.

[40] Angelucci A, Bullier J (2003). Reaching beyond the classical receptive field of V1 neurons: horizontal or feedback axons?. J Physiol-Paris.

[41] Serre T, Kouh M, Cadieu C, Knoblich U, Kreiman G (2005). A theory of object recognition: computations and circuits in the feedforward path of the ventral stream in primate visual cortex.. Massachusetts: Massachusetts Institute of Technology CBCL Paper.

[42] Gabor D (1946). Theory of communication. Part 1: The analysis of information.. JIEE.

[43] Jones JP, Palmer LA (1987). An evaluation of the two-dimensional Gabor filter model of simple receptive fields in cat striate cortex.. J Neurophysiol.

[44] Kusiak A, Chung Y (1991). GT/ART: Using Neural Networks To Form Machine Cells..

[45] Fei-Fei L, Fergus R, Perona P (2007). Learning generative visual models from few training examples: An incremental bayesian approach tested on 101 object categories.. Computer Vision and Image Understanding.

[46] Fergus R, Perona P, Zisserman A (2003). Object class recognition by unsupervised scale-invariant learning IEEE, Vol. 2. p.. II–264–II-271 vol.

[47] Weber M, Welling M, Perona P (2000). Unsupervised learning of models for recognition.. Computer Vision-ECCV.

[48] Huang Y, Huang K, Tao D, Tan T, Li X (2011). Enhanced Biologically Inspired Model for Object Recognition.. IEEE transactions on systems, man, and cybernetics Part B, Cybernetics: a publication of the IEEE Systems, Man, and Cybernetics Society.

[49] Wilcoxon F (1945). Individual comparisons by ranking methods.. Biometrics Bulletin.

[50] Massey FJ (1951). The Kolmogorov-Smirnov test for goodness of fit.. Journal of the American Statistical Association.

[51] Brainard DH (1997). The psychophysics toolbox.. Spatial vision.

[52] Pelli DG (1997). The VideoToolbox software for visual psychophysics: Transforming numbers into movies.. Spatial vision.

[53] Kleiner M, Brainard D, Pelli D, Ingling A, Murray R (2007). What's new in Psychtoolbox-3.. Perception.

[54] Logothetis NK, Pauls J, Poggio T (1995). Shape representation in the inferior temporal cortex of monkeys.. Current Biology.

